# Pediatric musculoskeletal ultrasound of the elbow: a pictorial review

**DOI:** 10.1007/s00247-026-06582-6

**Published:** 2026-03-20

**Authors:** Hamza Alizai, Matthew Hammer, Jennifer Nicholas

**Affiliations:** 1 Department of Radiology, Texas Scottish Rite Hospital for Children, Dallas, TX USA; 2 Department of Radiology, Children’s Hospital of Philadelphia, Philadelphia, PA USA; 3 UT Southwestern Medical Center, University of Texas Southwestern School of Medicine, Dallas, TX USA; 4 Cincinnati Children’s Hospital Medical Center, University of Cincinnati School of Medicine, 3333 Burnet Avenue, Cincinnati, OH 45229 USA

**Keywords:** Elbow, Ultrasound, Pediatric, Musculoskeletal

## Abstract

The elbow is a complex joint consisting of three separate articulations: ulnotrochlear, radiocapitellar, and proximal radioulnar. Collectively, these allow movements including flexion, extension, supination, and pronation. The developing pediatric elbow can be challenging to assess due to the predominance of cartilaginous epiphyseal and apophyseal structures, which are not evident on radiographs. Although MRI provides a comprehensive assessment of elbow anatomy, it can also be technically challenging. In a supine position with the arm to the side, the off-center position of the elbow can lead to decreased image quality due to magnetic field inhomogeneity with low signal-to-noise ratio (SNR) and poor fat suppression. Positions that attempt to bring the elbow to the MR iso-center can be uncomfortable in children suffering from elbow pain, leading to motion artifact. Furthermore, many of the soft tissue pathologies in the elbow are dynamic in nature and are best assessed using dynamic maneuvers. Ultrasound (US) is an ideal imaging modality for both static and dynamic assessment of the pediatric elbow joint and its soft tissue stabilizers. Additionally, comparison with the US of the contralateral elbow can serve as an internal control in the assessment of skeletally immature patients. In this narrative review, we describe the ultrasound technique for evaluating the pediatric elbow, the normal sonographic anatomy, and the common pathologies that may be encountered in practice.

## Introduction

The elbow is an anatomically and functionally complex joint consisting of three separate articulations: ulnotrochlear, radiocapitellar, and proximal radioulnar, which collectively allow flexion, extension, supination, and pronation. Elbow injuries are among the most common acute pediatric musculoskeletal conditions [1], particularly in children participating in overhead sports [2]. However, imaging assessment of the elbow in skeletally immature children is complex due to the primarily cartilaginous joint anatomy. The secondary ossification centers develop in a predictable course: the capitellum is the first to develop, followed by the radial head, medial epicondyle, trochlea, olecranon, and lateral epicondyle.

The pediatric elbow’s unossified cartilaginous structures and soft tissue anatomy cannot be adequately assessed radiographically. Although MRI enables a comprehensive assessment of the elbow joint, the need for anesthesia in children may pose a logistical challenge. Obtaining high-quality MRI can also be technically challenging due to the inherent anatomy of the elbow. In a supine position with the arm to the side and supinated, the off-center position of the elbow can lead to decreased image quality due to magnetic field inhomogeneity with low signal-to-noise ratio (SNR) and poor fat suppression. Positions that attempt to bring the elbow to the MR iso-center, such as prone with the arm in elevation and pronation of the elbow joint (superman position), can be uncomfortable in children suffering from elbow pain and lead to motion artifact. Moreover, many of the soft tissue pathologies in the elbow are dynamic in nature and are best assessed using dynamic maneuvers. Ultrasound can provide a rapid static, dynamic, and stress assessment of the elbow joint’s cartilage, tendons, ligaments, and osseous structures [3]. Furthermore, comparison with the US of the contralateral elbow can serve as an internal control in the assessment of skeletally immature patients. In patients with orthopedic hardware, ultrasound enables assessment of structures adjacent to hardware without the metal artifact that often limits MRI.

## Pediatric elbow: technique

Linear array high-frequency transducers (at least 12 MHz), including the compact linear array (hockey-stick) transducer, are optimal due to the higher resolution and relatively shallow anatomy of the elbow. The patient can be imaged while seated with the elbow positioned on an examination table across from the sonographer or while semi-supine [9]. Very young children may be examined while in the caregiver’s lap.

**Fig. 1:**
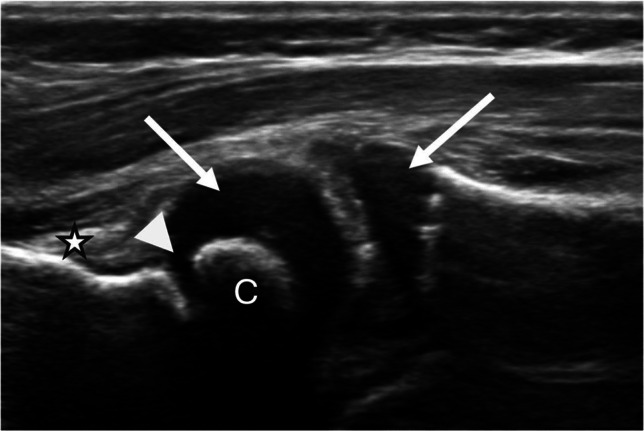
Normal anterior radiocapitellar joint in a 2-year-old boy. The unossified epiphyseal cartilage of the radial head and capitellum (*arrows*) is discernible. The linear hypoechoic physis (*arrowhead*) separates the epiphysis from the metaphysis. The echogenic fat pad of the anterior joint recess (*star*) is also visible

Figure 1 demonstrates the normal structures in the skeletally immature radiocapitellar joint of a 2-year-old.

The cortex of the bone is a smooth linear specular reflector and, therefore, appears as an echogenic line on ultrasound, while the physis appears as a hypoechoic line separating the metaphysis from the epiphysis [4]. The non-ossified epiphyseal cartilage is hypoechoic; the vascular channels within it appear as echogenic dots [3]. As the child matures, the articular hyaline cartilage remains hypoechoic. Muscle is predominantly hypoechoic with echogenic striations created by the epimysium and perimysium [3]. Fascia is hyperechoic on ultrasound. Tendons and ligaments demonstrate a hyperechoic fibrillar pattern, with tendons being more echogenic than ligaments. As tendons comprise highly organized fibrils, an ultrasound beam insonating the tendon at an angle other than 90° may reflect away from the transducer, leading to an artificially hypoechoic appearance of the tendon (anisotropy). It is important to recognize and correct for anisotropy to avoid incorrect perception of tendon pathology. Nerves have a honeycomb (fascicular) pattern in the short axis and a tram-track pattern in the long axis [3]. The nerve fascicles are hypoechoic, and the surrounding connective tissue, including the perineurium and epineurium, is echogenic.

For a structured evaluation, the elbow can be divided into four anatomic compartments (Table 1). All structures within each compartment should be examined in short and long axes for a comprehensive examination. Generally, skeletally immature athletes more frequently suffer from injuries to the physis and apophysis [5]. As skeletal maturity is reached, the injury patterns shift to injury of the ligaments, tendons, bone, and cartilage [5]. Ultrasound of the contralateral (asymptomatic) elbow can help differentiate between pathology and normal anatomic variations.

**Table 1:** Important elbow structures by compartment

Compartment	Key structures	Pathologies
Medial	Ulnar collateral ligament Common flexor tendon Ulnohumeral joint Ulnar nerve	UCL injury Medial epicondylar apophysitis Ulnar neuritis
Lateral	Lateral ligament complex Common extensor tendon Radiocapitellar joint	Osteochondral lesion Ligament injury Lateral epicondylitis
Anterior	Biceps and brachialis tendons Radial nerve and median nerve Anterior joint recess	Biceps tendinosis/tear Radial or median neuritis Effusion, synovitis, intraarticular bodies Distal humerus epiphyseal separation
Posterior	Triceps Olecranon bursa Posterior joint recess	Triceps tendinosis/tear Olecranon bursitis Effusion, synovitis, intraarticular bodies

A video demonstration of the sonographic examination of the elbow, illustrating appropriate probe positioning and normal sonographic anatomy, is available through the following link to a recent SPR workshop conducted by the authors of this review: https://myedu.spr.org/p/SPRMSK24PedRad.

### Ultrasound of the medial elbow

The key structures in the medial elbow include the ulnohumeral articulation, the ulnar collateral ligament (UCL), the common flexor tendon, and the ulnar nerve.

Ulnar collateral ligament injuries are most common in adolescents, with 70% of the injuries occurring in patients aged 10–19 [6]. UCL is the primary restraint against valgus stress on the elbow. In children who participate in overhead sports, particularly baseball, the forces generated can exceed the physeal or UCL’s failure strength [2]. Repetitive stress-related epicondylar apophysitis may present with physeal widening, apophyseal irregularity, and hyperemia. Ultrasound has been shown to have a high positive predictive value for all medial elbow pathology [7]. A recent study of pediatric UCL injuries found that apophyseal avulsion injuries are seen in younger adolescents (mean age 10.3 for cartilaginous avulsion, 10.6 for bony avulsion) and central ligament injuries in older adolescents (mean age 14.2) [8].

**Fig. 2:**
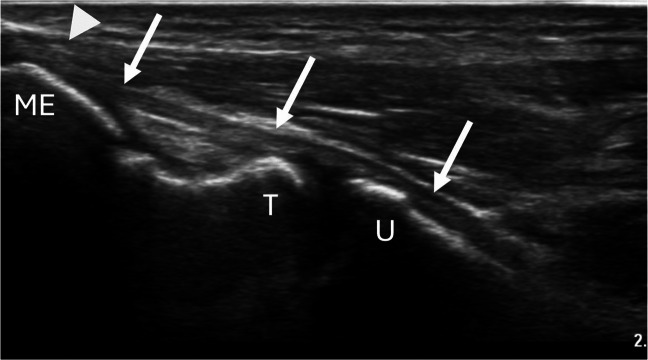
Normal elbow of a 14-year-old male baseball pitcher. Key bony landmarks for sonographic evaluation of the ulnar collateral ligament include the medial epicondyle (*ME*), the shallow U-shaped humeral trochlea (*T*), and the sublime tubercle of the ulna (*U*). The UCL appears as a fibrillar, echogenic band extending from the undersurface of the medial epicondyle to the sublime tubercle (*white arrows*). The common flexor tendon lies superficial to the UCL, attaching at the medial epicondyle (*arrowhead*)

Anatomically, the UCL consists of three bundles: anterior, posterior, and transverse. The anterior bundle, which extends from the medial epicondyle to the sublime tubercle of the ulna, is the most important restraint against valgus stress [2]. Sonographic examination of the UCL is performed with the arm externally rotated, the elbow flexed, and the wrist supinated [3]. On ultrasound, the UCL appears as a fan-shaped echogenic structure extending from the undersurface of the medial epicondyle to the sublime tubercle. Figure 2 illustrates normal UCL and common flexor tendons in a 14-year-old male baseball player.

**Fig. 3:**
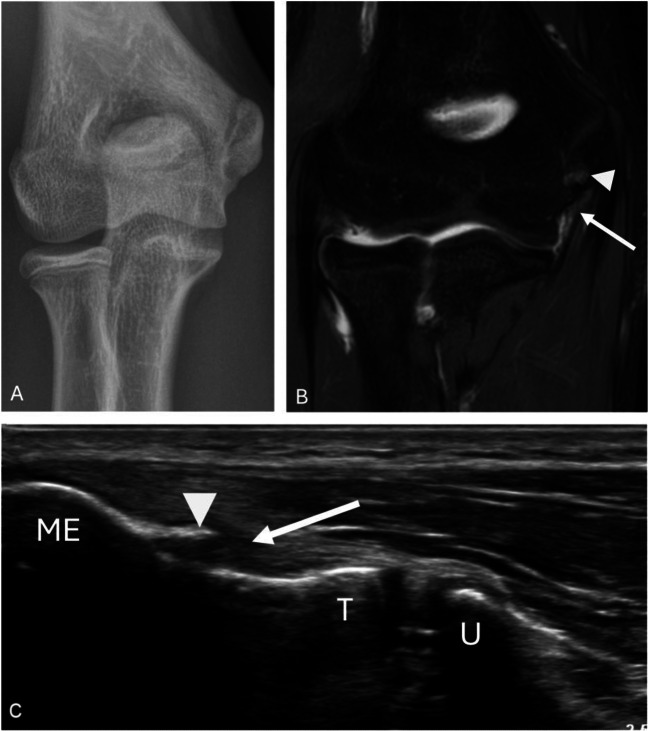
A 13-year-old male baseball pitcher presents with right elbow pain. **A** Coronal radiograph of the elbow demonstrates no acute osseous abnormality. **B** Coronal short tau inversion recovery MRI sequence shows a nondisplaced avulsion fracture of the medial epicondyle (*arrowhead*) and a heterogeneous, thickened proximal anterior band of the ulnar collateral ligament with increased intrinsic signal (*arrow*). **C** Follow-up elbow ultrasound obtained 2 months later demonstrates healing and coalescence of the nondisplaced avulsion fracture (*arrowhead*) with persistent heterogeneity of the proximal ulnar collateral ligament (*arrow*)

The earliest sonographic abnormality of the UCL with chronic repetitive valgus stress may include thickening when compared to the contralateral (unaffected) side [9]. Tearing of the UCL manifests with a hypoechoic or anechoic cleft disrupting the ligament fibers, which may occur at the proximal attachment, the mid-substance, or the distal attachment and may include osseous avulsions, particularly at the medial epicondylar attachment. Figure 3 demonstrates an osseous avulsion injury in a 13-year-old male baseball pitcher.

**Fig. 4:**
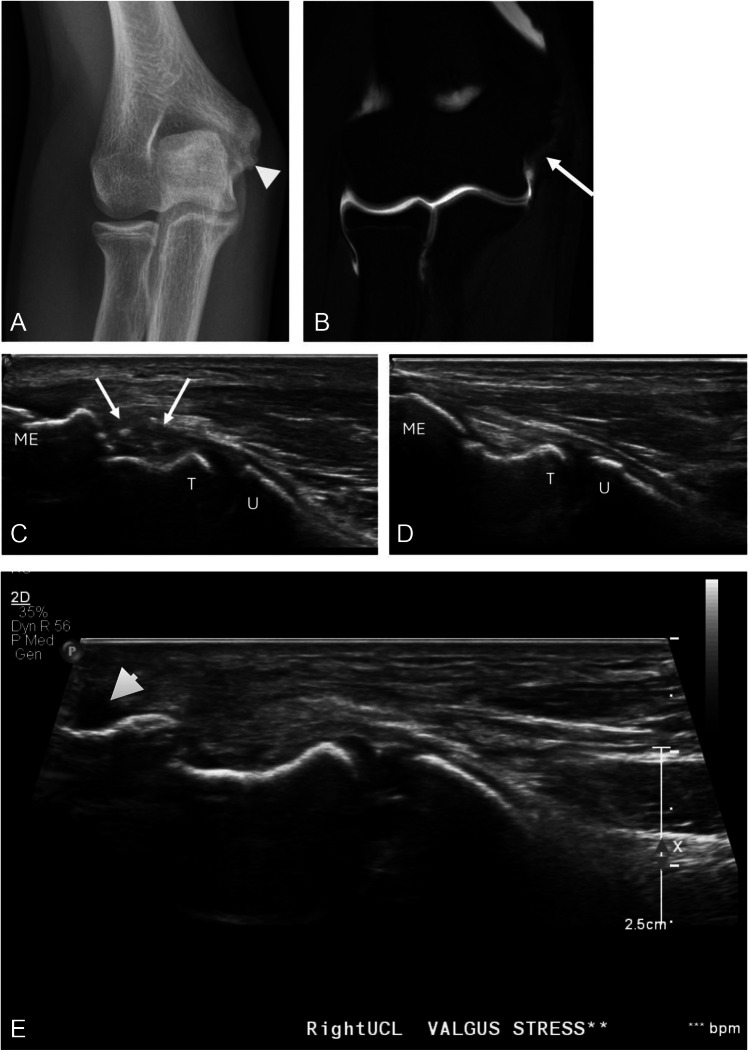
A 14-year-old male baseball pitcher presented with recurrent right elbow pain 1 year after surgical fixation of a medial epicondylar avulsion fracture. **A** Coronal radiograph demonstrates osseous remodeling of the medial epicondyle without evidence of an acute avulsion. **B** Coronal T1 fat-saturated MR arthrogram image shows a heterogeneous, thickened proximal UCL without a discrete tear (*arrow*). **C** Stress ultrasound of the elbow, performed at the surgeon’s request, again demonstrates osseous remodeling of the medial epicondyle. The proximal ulnar collateral ligament appears thickened with internal echogenic sutures but without a definite tear. **D** US of the normal contralateral elbow for comparison. No asymmetric increase in ulnohumeral joint gapping during valgus stress maneuvers. **E** Cine clip obtained during dynamic stress evaluation reveals a small focal partial tear of the common flexor tendon attachment (*arrowhead*)

Functional insufficiency of the UCL can be diagnosed using sonographic valgus stress examination, which shows increased ulnohumeral gapping (distance from the trochlea to sublime tubercle) [10]. This examination can be performed during application of manual valgus stress or with the addition of wrist weights, inducing valgus stress at the elbow. Commercial devices for the application of standardized joint stress [11] are also available, but not widely utilized in clinical practice. Although measurement thresholds for ulnohumeral gapping have been proposed in the adult population [3], these studies have focused on adult patients. Comparison with the unaffected side offers the best reference standard for this assessment in children. Figure 4 includes static images and accompanying cine clip demonstrating a valgus stress examination in a 14-year-old male baseball pitcher presenting with recurrent elbow pain 1 year after surgical fixation of a medial epicondylar avulsion fracture.

The common flexor tendon (CFT) of the elbow comprises the flexor carpi radialis, palmaris longus, flexor carpi ulnaris, flexor digitorum superficialis, and the pronator teres. It attaches to the medial epicondyle and provides secondary restraint to valgus stress [4]. Common flexor tendon injuries are uncommon in children, which may lead to symptoms initially misattributed to UCL pathology. Abnormalities of the CFT can, however, be seen in the setting of significant overuse or traumatic elbow injuries. The spectrum of tendon abnormalities may include thickening, heterogeneity, and hyperemia in the setting of tendinosis to partial or complete discontinuity of the tendon fibers. In skeletally immature children, avulsion injuries of the medial epicondylar ossification centers are more common than tendon injuries due to apophyseal vulnerability.

**Fig. 5:**
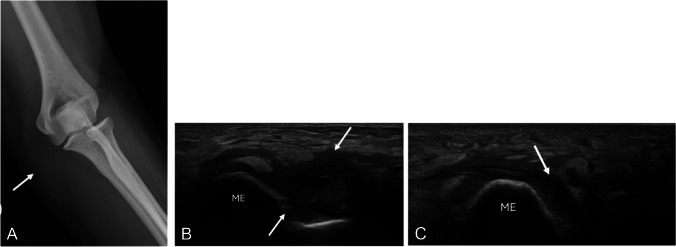
A 16-year-old male presented with medial elbow pain, numbness, and tingling following a football injury. **A** Radiograph of the elbow demonstrates marked medial soft tissue swelling without evidence of fracture. **B** Long-axis ultrasound image of the affected elbow reveals a large fluid-filled gap (*between arrows*) extending from the subcutaneous soft tissues to the medial epicondyle, consistent with complete tears of both the common flexor tendon and the ulnar collateral ligament. **C** Short-axis ultrasound image shows obscuration of the ulnar nerve, which is surrounded by fluid and hematoma. The patient subsequently underwent surgical repair of the common flexor tendon and ulnar collateral ligament reconstruction

Figure 5 demonstrates a complete tear of the CFT and the UCL in a 16-year-old who suffered an injury during a high school football game.

**Fig. 6:**
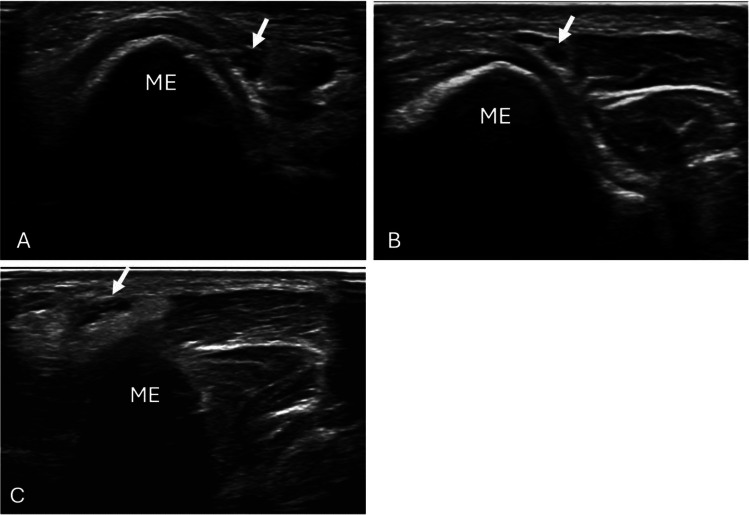
Axial ultrasound images of the medial elbow in a 16-year-old male football player with a snapping sensation and tingling at the medial elbow. Images obtained at approximately 15° (**A**), 30° (**B**), and 45° (**C**) of elbow flexion demonstrate anterior translation of the ulnar nerve (*arrows*) from posterior to the medial epicondyle (*ME*), confirming dynamic instability. The ulnar nerve shows abnormal morphology with fascicular enlargement and decreased echogenicity

Ulnar nerve entrapment (cubital tunnel syndrome) is uncommon in children [12] but has been reported with excessive video game play and overuse during overhead sports, as well as with traumatic injuries to the medial elbow. Ulnar neuropathy at the elbow can also be seen in some cases of ulnar nerve instability [13, 14]. Sonographic evidence of neuropathy may include nerve enlargement, decreased echogenicity, and loss of normal fascicular (honeycomb) morphology. Figure 6 demonstrates the instability of a morphologically abnormal ulnar nerve in a 16-year-old football player.

### Ultrasound of the lateral elbow

The key structures in the lateral elbow include the radiocapitellar joint, the common extensor tendon, and the lateral collateral ligament complex (Joint anatomy in a skeletally immature patient is illustrated in Fig. 1).

**Fig. 7:**
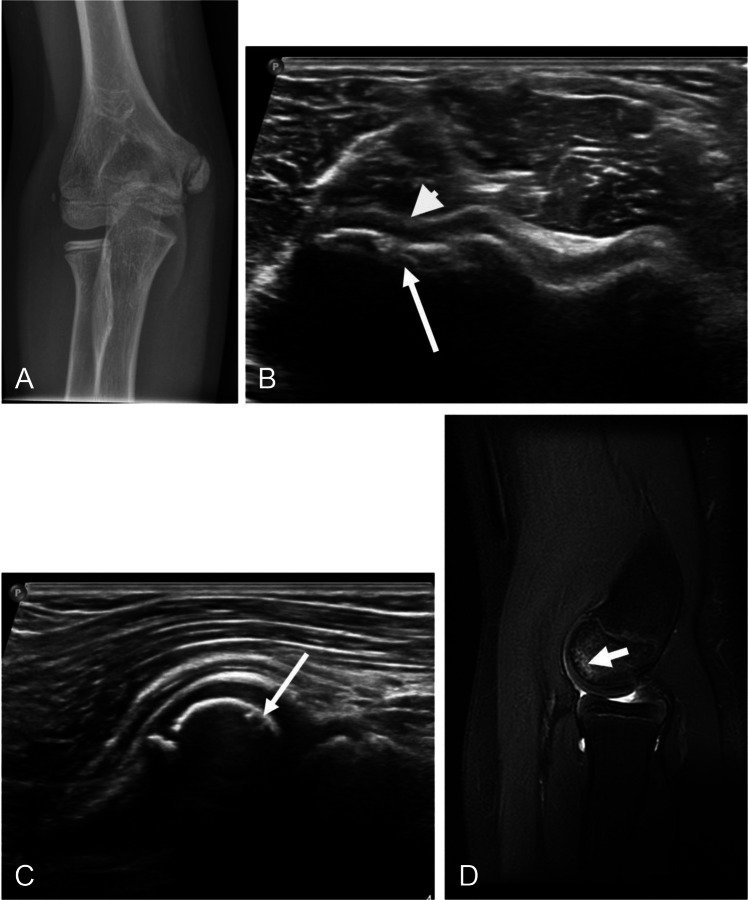
A 10-year-old male baseball player with chronic elbow pain. **A** Frontal radiograph of the elbow appears normal. **B** Axial and **C** long-axis ultrasound images demonstrate focal subchondral irregularity of the capitellum (*arrows*). Intact overlying hypoechoic hyaline articular cartilage (*arrowhead*). **D** Sagittal short tau inversion recovery MR image confirms subchondral marrow signal abnormality consistent with osteochondritis dissecans (*arrow*)

Osteochondritis dissecans (OCD) of the elbow is seen in overhead athletes and is considered an overuse injury due to repetitive valgus stress. It most commonly involves the capitellum with a varying spectrum of osseous resorption, fragmentation, and articular cartilage involvement [3]. Sonographic assessment for OCD has been found to correlate well with MRI and may have utility in the diagnosis of instability of these lesions [15, 16]. Osteochondral abnormalities on ultrasound may include decreased echogenicity of the articular cartilage, subchondral bone irregularity, or fragmentation of cartilage or bone. Figure 7 demonstrates an OCD in a 10-year-old male baseball pitcher with chronic elbow pain.

**Fig. 8:**
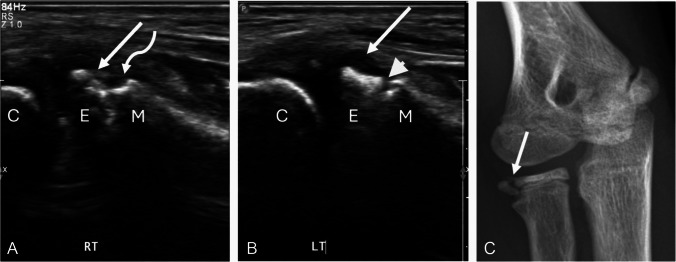
An 11-year-old football player presented 3 months after a helmet impact to the right elbow sustained during a game. The initial clinical concern was a ligamentous injury, prompting an ultrasound evaluation. Sonography of the lateral elbow demonstrates irregularity of the radial head articular cartilage and subchondral bone (*arrow*) as well as adjacent metaphyseal irregularity (*curved arrow*). Normal anatomy is seen on the contralateral left (*LT*) side with bony landmarks: capitellum (*C*), radial epiphysis (*E*), and radial metaphysis (*M*). The articular cartilage (*arrow*) and physis (*arrowhead*) appear normal. Subsequent radiographs of the right elbow demonstrate a radial head fracture extending to the physis

Ultrasound has been shown to have high sensitivity and specificity for pediatric elbow fractures [17]. Sonographic evaluation of fractures may reveal cortical disruption or secondary signs such as hemarthrosis. Fracture detection with US has been predominantly performed in the context of point-of-care ultrasound (POCUS) rather than traditional radiology practice. It may be useful in cases where the clinical suspicion for a fracture is high, but the radiographs are normal or equivocal. Figure 8 shows a radial head fracture in an 11-year-old male football player who presented 3 months after the initial injury.

The common extensor tendon comprises contributions from the extensor carpi radialis brevis and longus, extensor digitorum, extensor carpi ulnaris, and extensor digiti minimi. Medial-sided elbow injuries predominate in skeletally immature elbows due to apophyseal vulnerability; however, lateral-sided pathology becomes more frequent after physeal closure. Lateral epicondylitis (tennis elbow) is common in adults but can be infrequently seen with significant overuse in athletic adolescents. The spectrum of abnormalities may include thickening, heterogeneity, and hyperemia in the setting of tendinosis to partial or complete discontinuity of the tendon fibers.

The lateral collateral ligament complex provides restraint against varus stress and consists of the radial collateral ligament (RCL), the annular ligament, and the lateral ulnar collateral ligament (LUCL). The RCL originates at the lateral epicondyle and inserts on the annular ligament. The annular ligament encircles the radial head and attaches on the anterior and posterior margins of the radial notch on the ulna, keeping the radial head in place during supination/pronation. This is often injured during radial head subluxation injuries (nursemaid’s elbow). The LUCL originates at the lateral epicondyle and inserts on the supinator crest of the ulna and functions as the primary restraint against posterolateral instability. Injuries of the lateral ligamentous complex are uncommon in children but may be seen in the setting of major trauma [18].

### Ultrasound of the posterior elbow

**Fig. 9:**
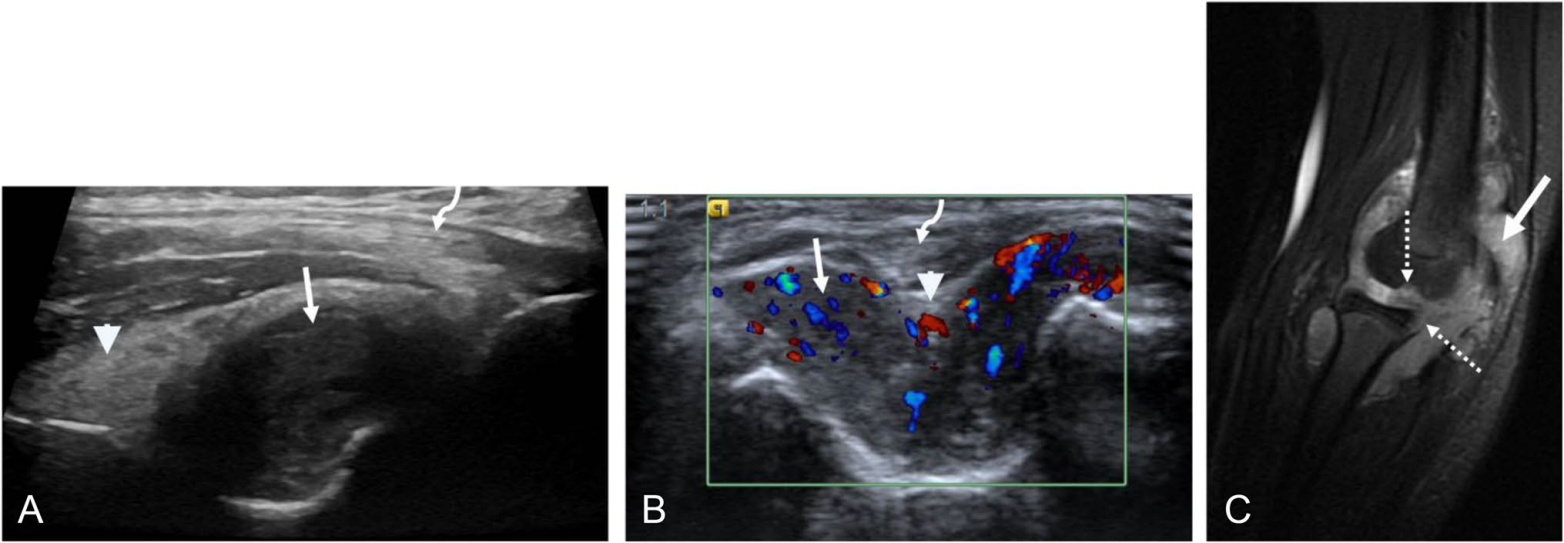
An 8-year-old girl with a known diagnosis of juvenile idiopathic arthritis presenting with elbow swelling. Long-axis (**A**) and axial (**B**) ultrasound images of the posterior elbow demonstrate the U-shaped concavity of the olecranon fossa filled with a hypoechoic, hyperemic region of synovitis (*arrow*). The posterior fat pad is elevated (*arrowhead*). The triceps tendon (*curved arrow*) appears echogenic and fibrillar, seen superficial to the posterior joint recess as it extends toward its insertion on the olecranon. **C** MR sagittal T2 fat saturation sequence in the same patient demonstrating the extent of severe synovitis (*arrow*) and erosive changes (*dashed arrows*) at the radiocapitellar articulation

The key structures in the posterior elbow include the posterior joint recess, the triceps muscle, the tendon, and the olecranon bursa. Sonographic examination of the posterior elbow is best performed with the elbow in a flexed position [4]. The olecranon fossa is easily discernible as a concavity filled with the echogenic posterior fat pad of the elbow. Joint effusions, hemarthrosis, and synovitis can all result in elevation of the posterior fat pad. Synovitis appears as noncompressible hypoechoic to isoechoic synovial thickening with increased vascularity on Doppler imaging. Figure 9 shows severe elbow synovitis in an 8-year-old with an established diagnosis of juvenile idiopathic arthritis. Effusions and hemarthrosis are both compressible and avascular. Simple effusions are anechoic, whereas hemarthrosis shows variable, often heterogeneous, echogenicity, becoming more echogenic as clotting develops.

**Fig. 10:**
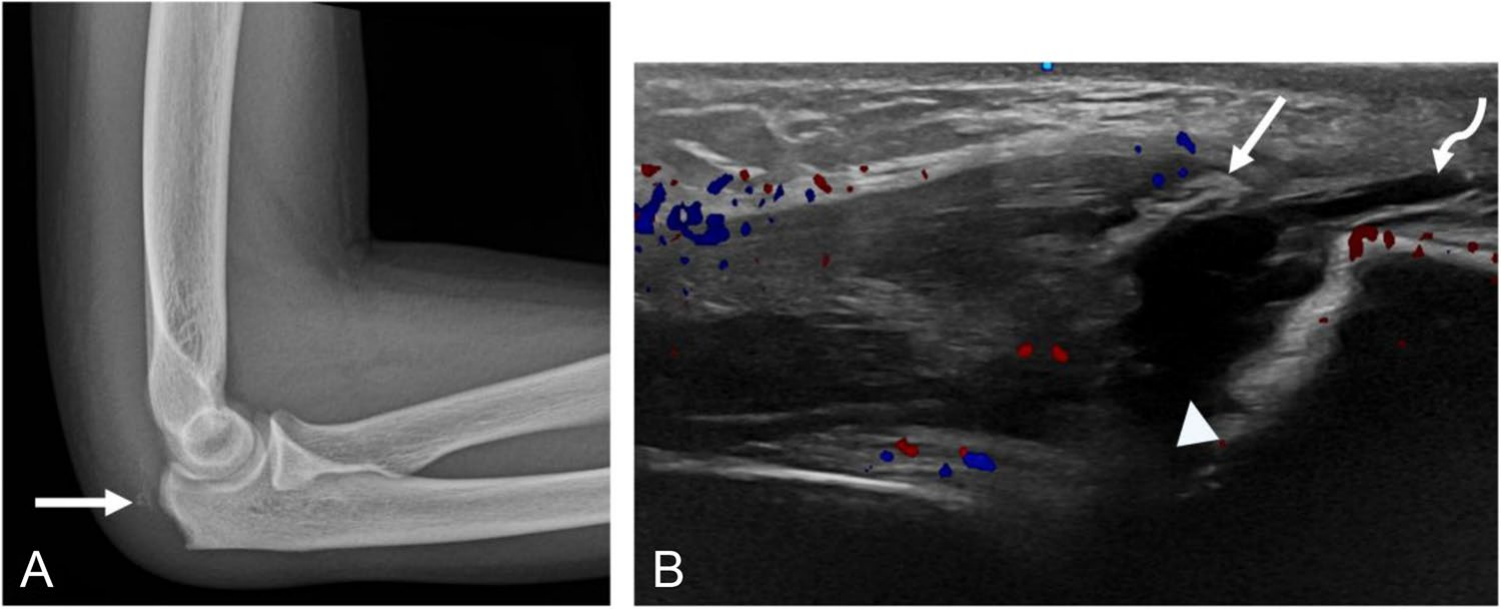
A 15-year-old girl with acute elbow pain after hearing a “pop” while throwing a football. **A** Lateral radiograph of the elbow shows an avulsed ossific fragment (*arrow*) dorsal to the olecranon. **B** Ultrasound demonstrates a complete retracted tear of the triceps tendon with a fluid- and blood-filled gap (*arrowhead*) and the avulsed ossific fragment at the olecranon insertion (*arrow*). Note the associated fluid distention of the olecranon bursa (*curved arrow*)

The triceps consist of three heads—medial, lateral, and long—of which the lateral and long heads converge to form a common tendon that inserts posterior to the insertion of the medial head on the olecranon [4]. The triceps tendon is superficial to the olecranon fossa and should be evaluated in both short-axis and longitudinal planes. Distal triceps tendinosis manifests with thickening of the tendon and decreased and heterogeneous echogenicity with or without hyperemia. Distal triceps tendon tears are uncommon in children and difficult to evaluate clinically due to pain and swelling. Ultrasound enables assessment of both the extent of discontinuity (partial vs complete tear) and the degree of retraction. Distal triceps injuries frequently result in avulsion fractures of the olecranon [4]. Figure 10 shows a full-thickness triceps tendon tear in a 15-year-old female patient with associated bony avulsion.

The olecranon bursa is located superficial to the distal triceps tendon and the olecranon process. Bursitis (inflammation of the bursa) manifests with fluid accumulation and synovial proliferation with or without hyperemia. Olecranon bursitis may develop due to trauma, infection, or the presence of systemic inflammatory conditions such as juvenile idiopathic arthritis [19].

### Ultrasound of the anterior elbow

The key structures in the anterior elbow include the anterior joint surfaces and recess, the biceps and brachialis myotendinous structures, and the median nerve.

**Fig. 11:**
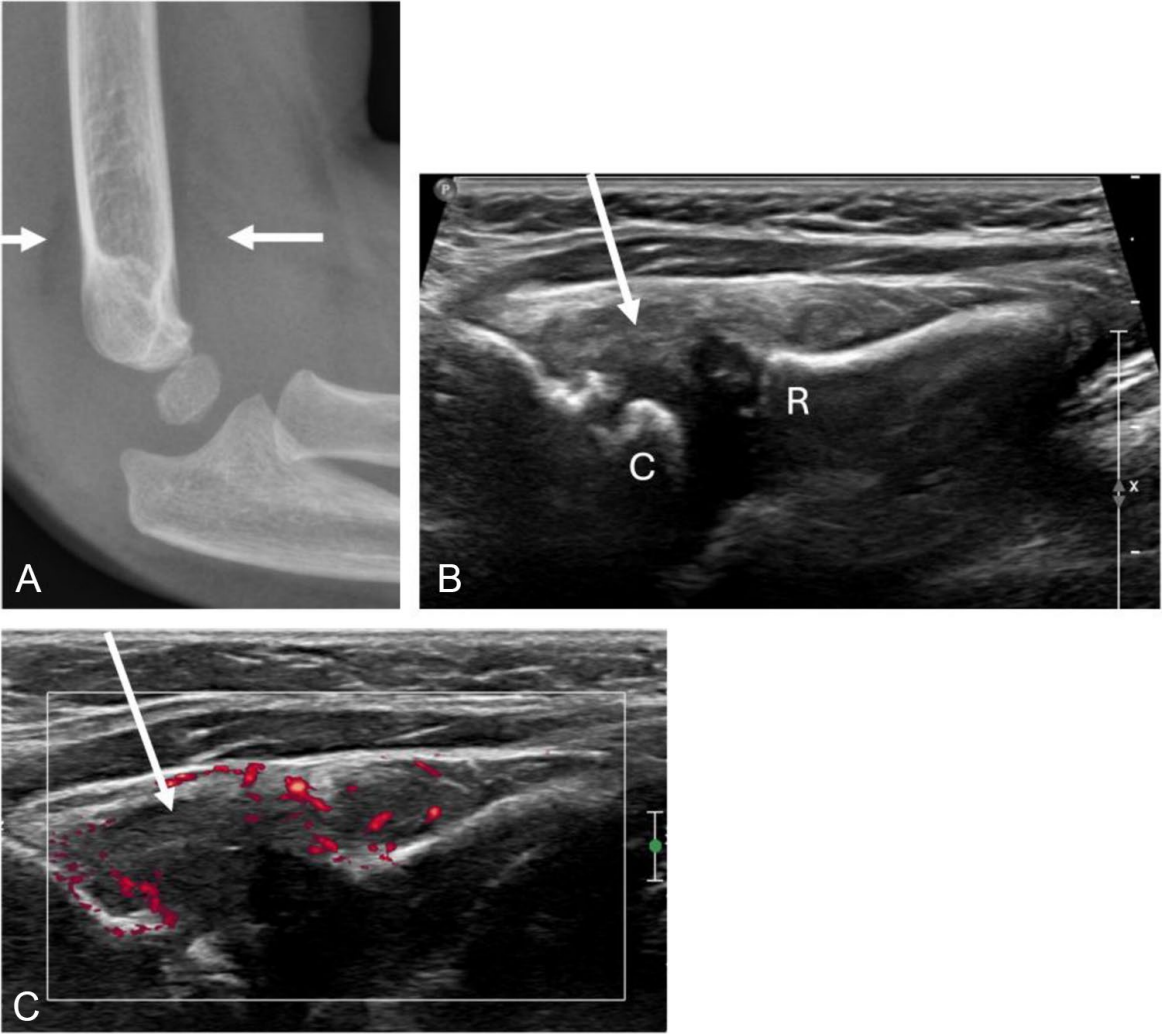
A 2-year-old girl with elbow swelling later diagnosed with oligoarticular juvenile idiopathic arthritis. **A** Lateral radiograph of the elbow shows elevation of the anterior and posterior fat pads (*arrows*), consistent with joint effusion. **B** Long-axis ultrasound of the anterior elbow at the level of the radiocapitellar articulation (*R*, radius; *C*, capitellum) demonstrates a hypoechoic region of synovitis (*arrow*) within the anterior joint recess, with associated hyperemia evident on Doppler imaging (**C**)

The anterior joint surfaces are best assessed in the long axis (coronal plane), which allows evaluation of the radiocapitellar and ulnotrochlear articular surfaces and the anterior elbow joint recess containing an echogenic fat pad. As previously mentioned, both joint effusion and synovitis can result in elevation of the elbow fat pads, and it is important to be able to distinguish between the two. Figure 11 shows synovitis in the anterior elbow joint recess in a 2-year-old with oligoarticular juvenile idiopathic arthritis.

**Fig. 12:**
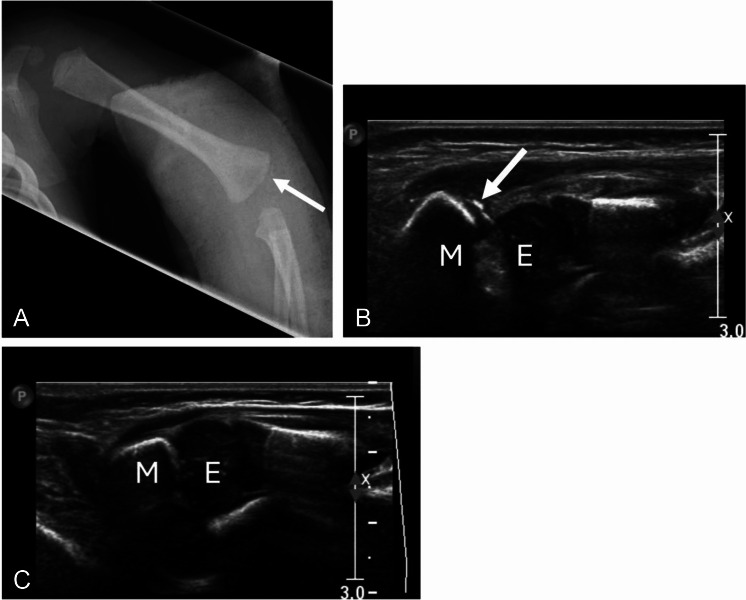
Distal humeral physeal separation in a 4-month-old girl. The patient presented with left elbow swelling and pain after a reported fall from a swing. Nonaccidental trauma was suspected. **a** Frontal radiograph shows medial translation of the proximal radius and ulna relative to the distal humerus, findings consistent with distal humeral physeal separation. **b** Longitudinal ultrasound image of the affected (*left*) radiocapitellar articulation demonstrates posterior displacement of the distal humeral epiphysis (*E*) relative to the metaphysis (*M*). Comparison image of the contralateral (*right*) elbow shows a normal radiocapitellar relationship. Also note a bucket-handle fracture of the lateral humerus visible on both the radiograph and the ultrasound (*arrow*)

Distal humerus epiphyseal separation is a rare injury, most commonly due to birth trauma in neonates or a fall in older children under 3 years of age. It is often missed completely on imaging or misdiagnosed as an elbow dislocation [20]. In children under 8 months of age, the absence of the capitellar ossification can make assessment of elbow joint alignment challenging on radiography. Radiographs in these patients will show posteromedial translation of the proximal radius and ulna relative to the distal humerus. Ultrasound can be used to confirm the separation of the humeral epiphysis from the metaphysis. Figure 12 shows distal humeral epiphyseal separation in a 4-month-old child who was accidentally dropped.

The brachialis and biceps muscles are both primary flexors of the elbow joint and are located between the brachioradialis laterally and the pronator teres medially. The biceps muscle is located superficial to the brachialis. It consists of two heads: the long head and the short head. The distal biceps tendon inserts on the radial tuberosity. Due to its oblique and rotational course, sonographic evaluation of the distal biceps tendon can be challenging; anisotropy is common, and thus, all apparent pathology should be scrutinized. Fortunately, pathology of the distal biceps and brachialis is exceedingly rare in the pediatric population.

The median nerve is easily identifiable as it is located medial to the brachial artery and extends distally between the humerus and the ulnar heads of the pronator teres, innervating the flexor musculature [4]. Median nerve pathology at the elbow is exceedingly rare in children as well as adults, but traumatic lacerations have been reported [21].

## Conclusion

Elbow pathology in children encompasses a range of traumatic, developmental, and overuse injuries, often influenced by the degree of skeletal immaturity. Ultrasound, combined with clinical evaluation, provides a rapid, comprehensive assessment of the elbow. Utilizing a compartment-based checklist approach ensures accurate systematic evaluation of all joint structures, while the contralateral elbow can serve as an effective internal control for developmental variations. Dynamic maneuvers further enhance accuracy by allowing real-time assessment of ligamentous laxity, tendon pathology, and ulnar nerve instability. In summary, adapting pediatric elbow ultrasound can facilitate early diagnosis of many of the conditions causing elbow pain in children and guide appropriate management.

## Data Availability

No datasets were generated or analysed during the current study.
